# Mechanism Understanding of the Role of Rare Earth Inclusions in the Initial Marine Corrosion Process of Microalloyed Steels

**DOI:** 10.3390/ma12203359

**Published:** 2019-10-15

**Authors:** Meng Tang, Kaiming Wu, Jing Liu, Lin Cheng, Xian Zhang, Yan Chen

**Affiliations:** 1 The State Key Laboratory of Refractories and Metallurgy, Hubei Province Key Laboratory of Systems Science in Metallurgical Process, Wuhan University of Science and Technology, Wuhan 430081, China; tangmeng@wust.edu.cn (M.T.); kaimingwu@wust.edu.cn (K.W.); zhangxian@wust.edu.cn (X.Z.); 2 Shanghai Aerospace Equipment Manufacturer, Shanghai 200245, China; chenlemi@126.com

**Keywords:** RE microalloyed steel, corrosion behavior, inclusion, first principle modeling, SEM, SVET

## Abstract

In this study, the corrosion behavior of rare earth (RE) microalloyed steels was first evaluated through potentiodynamic polarization tests and corrosion weight loss experiments, and then the corrosion morphologies were observed by scanning electron microscope (SEM). After immersion in a NaCl solution, the sulfides (or oxygen sulfides) dissolved preferentially, followed by corrosion at the boundary between the Fe matrix and oxides. Afterwards, the inclusions fell off as a whole, which promoted pitting nucleation. The first principle modeling demonstrated that the work functions of various kinds of inclusions descended in the following order: La_2_Zr_2_O_7_ > LaAlO_3_ > (La_2_O_3_ ≈ Fe ≈ La_2_O_2_S) > La_2_S_3_, which provided a theoretical explanation to the dissolution behaviors of inclusions. That is, inclusions containing sulfur tend to dissolve preferentially, whereas the oxides do not dissolve easily. Subsequently, the surface current distributions were detected by the scanning vibrating electrode technique (SVET), which provided more microscopic insight into the role of inclusions in the corrosion propagation. Results showed that the active sites of pitting nucleation accelerated the transverse propagation of corrosion. Finally, local corrosion spread to the whole surface as uniform corrosion.

## 1. Introduction

Corrosion in steels normally originates from local corrosion, and then propagates to the whole surface. The heterogeneous microstructural features, such as grain boundaries, second phases, dislocations, and inclusions, particularly sulfide inclusions, are the origin of local corrosion [1,2,3,4,5,6,7,8,9,10,11,12,13]. Therefore, many researchers have conducted research to elucidate the effects of inclusions on local corrosion to minimize their negative impacts [3,4,5,6,7,8,9,10,11,12,13].

For the past few years, research on rare earth (RE) microalloyed steel has attracted much attention [14,15,16]. First of all, the doping of RE elements can improve the comprehensive mechanical properties of microalloyed steels due to their effects on the purification of steel liquid, improvement of phase transformation, and modification of second phases, which has been investigated extensively [16,17,18,19,20,21,22,23,24,25,26]. Secondly, the addition of RE elements has an effect on corrosion properties of microalloyed steels by promoting the formation of passive corrosion product films [22,27,28,29], and modifying inclusions [25,30]. However, only a few studies have investigated the effect of RE microalloying on the corrosion behavior. RE elements, acting as microalloyed elements, can modify the type and property of inclusions due to their great affinity for oxygen and sulfur [8,30,31,32,33,34,35]. It has been reported that, with the doping of RE elements, the main types of inclusions change into RE sulfides, RE oxides, and RE oxygen sulfides [14,24,34,35,36,37,38]. Moreover, the sizes of RE-doped inclusions are much smaller than those of normal inclusions [23,24,35], and inclusions change from irregular shapes to spherical or spheroid [35,39,40]. However, these studies provided only superficial descriptions and lacked in-depth analyses. Relatively few researches have elaborately studied the effects of RE-doped inclusions on the corrosion behavior of steels [35,36,37,41].

Ha et al. [36] engaged in the study of the corrosion behavior of 25% Cr duplex stainless steel after the doping of RE elements. According to the statistical results, pitting originated at the matrix (Cr, Mn, RE)–oxides interface, and the quantity rather than the size of inclusions had a great effect on the corrosion resistance of this stainless steel. Jeon et al. [35] also reported that the pitting nucleation originated at the boundary between the matrix and RE oxides, and the micro crevices around inclusions spread into the matrix with no change in the RE oxides. However, the behavior was not the same for microalloyed steel compared with stainless steels. Liu et al. [37] recently revealed that the pitting corrosion of RE microalloyed steel was originated from the (RE)_2_O_2_S-(RE)_x_S_y_ inclusion for its lower potential relative to the matrix. However, the conclusions about the corrosion behaviors of the inclusions are not comprehensive from a statistical viewpoint. Moreover, the effect of RE-doped inclusions on the entire corrosion process, from pitting initiation to propagation, has not been clearly illuminated. Hence, it is worth investigating the effect of RE inclusions on the corrosion process of RE microalloyed steels.

Therefore, in this present work, the role of RE inclusions in the initial corrosion process of microalloyed steels was investigated elaborately. By using first principle modeling and the scanning vibrating electrode technique (SVET), a more in-depth mechanism understanding about the role of RE inclusions in the corrosion process was established theoretically and experimentally. Furthermore, the corrosion resistance of microalloyed steels with different deoxidized methods was comparatively studied.

## 2. Experimental Procedure

### 2.1. Materials Preparation

RE microalloyed steels employed in this study were smelted in a 50 kg vacuum induction furnace (KZG-50, Henan Cooler Instrument Technology Limited Company, Zhengzhou, China) by adding the alloys (Wuhan Iron and Steel Group Corporation, Wuhan, China) to the metal stream during tapping [42], and subsequently hot rolled to a thickness of 20 mm, followed by room temperature cooling. In the smelting process, different deoxidization methods (i.e., Al deoxidization and Ti/Zr deoxidization) were adopted for comparative study, and mischmetal (La and Ce) was added as microalloyed elements. The chemical compositions of the steels were analyzed by an inductive coupled plasma-mass spectrometer (ICP-MS, Thermo X Series II, Thermo Fisher Scientific, Waltham, Mass, America), and results are shown in Table 1.

The steel plates were cut into coupons of 20 × 20 × 5 mm^3^ along the rolling direction to meet the test requirements. For electrochemical tests, the specimens were embedded in epoxy resin (E-44, Solid Adhesive Electronic Technology Limited Company, Shenzhen, China) leaving an area of 4 cm^2^ as a working electrode surface, while the other end of the specimens was connected to copper wire for electrical connection. Afterwards, specimens were gradually wet ground to a 2000 grit finish by abrasive paper. Furthermore, the specimens were polished to a 0.5 µm finish for SEM (Evo, MA10, Zeiss, Germany) observations and SVET (Versascan, Ametek Limited Company, Polly, Pennsylvania, America) measurements. Finally, all the specimens were degreased with acetone (Sinopharm Chemical Reagent Limited Company, Ningbo, China), rinsed with deionized water, and dried by a compressed hot air flow.

### 2.2. Corrosion Rate Measurements

#### 2.2.1. Potentiodynamic Polarization Tests

A Zahner Zennium electrochemical workstation (E4, Zahner Company, Kronach, Germany) was used to perform the potentiodynamic polarization tests. A three-electrode cell was used, which includes a saturated calomel (SCE) reference electrode, a counter electrode of platinum plate with a dimension of 4 mm^2^, and a specimen as the working electrode. The test solution was 3.5 wt.% NaCl (Sinopharm Chemical Reagent Limited Company, Shanghai, China) with a volume of 500 mL, which stayed at 25 ± 1 °C during the tests. Potentiodynamic scans started at −300 mV_vs.OCP_ until the current density increased to 1 mA·cm^−2^. The scan rate was 0.333 mV s^−1^. For better reproducibility, all electrochemical measurements were repeated more than three times.

#### 2.2.2. Weight Loss Experiments

To ensure the reproducibility of the results, more than three specimens were prepared in the immersion tests. The specimens were weighed with a digital balance (SQP, Sartorius Scientific Instrument Limited company, Göttingen, German) with an accuracy of 0.0001 g before immersion. Then, the specimens were immersed in a 3.5 wt.% NaCl solution with a volume of 1 L at a bath temperature of 25 ± 1 °C for 1, 2, 5, 10, 20, and 50 days, to evaluate the corrosion rate for a prolonged period. At each interval, the corroded specimens were extracted and the corrosion products were removed with an HCl solution of specific composition (500 mL HCl + 500 mL H_2_O + 10 g C_6_H_12_N_4_) for approximately 3 min to remove the corrosion products. Afterwards, the specimens were ultrasonically rinsed (KQ-300DE, Kunshan Ultrasonic Instrument Limited Company, Kunshan, China) in ethanol, dried by hot air flow, and reweighed to evaluate the weight loss.

### 2.3. Microstructure Characterization

The statistical characteristics of the inclusions were identified using a scanning electron microscope (SEM, Evo, MA10, Zeiss, Germany), equipped with an energy dispersive spectrometer (EDS, Genesis 2000, Zeiss, Germany). Additionally, the micro-morphologies of inclusions were observed by a Zeiss-Auriga SEM.

### 2.4. First Principle Modeling

A commercial software (version 5.4, University of Vienna, Vienna, Austria) named Vienna ab initio simulation package (VASP) was used for the first principle calculation [43,44]. The modeling method was the projector augmented wave (PAW) method [45] and generalized gradient approximation (GGA) within the Perdew–Burke–Ernzerhof (PBE) exchange-correlation function [46]. Based on experimental results, the cut-off energy was set at 400 eV, and the parameter, ISIF, when modelling in VASP software, was set to 3 to obtain the optimized lattice constants of all inclusions. A slab model without relaxation with a vacuum layer of about 15 angstroms in thickness was utilized to calculate the averaged vacuum static potential and Fermi energy of different low-indexed crystallographic planes of the inclusions to acquire their work functions and surface energies. The Brillouin zone for all the slab models of approximately 30 angstroms in total thickness was sampled by a 7 × 7 × 1 k-mesh grid generated by the Monkhorst–Pack scheme.

### 2.5. SVET Measurements

To analyze the microelectrochemical activity on the surface, a scanning vibrating electrode technique (SVET, Versascan, Ametek, Polly, Pennsylvania, America) was utilized to detect the localized distribution of the current density on the metal surface [1,47,48,49]. The sealed electrochemical specimen was fixed in the sample holder in the direction normal to the Pt-Ir microelectrode, which has a diameter of approximately 5 μm. The vibrating amplitude (*d*) and frequency (*f*) of the microelectrode was 30 μm and 80 Hz in the normal direction, respectively. The test solution was a 0.5 wt.% NaCl with a volume of about 250 mL. During the experiments, the potential differences between the vibration peak and valley, Δ*E*, were detected by an electrometer incorporated in this equipment. Therefore, the local distribution of current density could be acquired through the equation *I* = −Δ*E*/*R*, where *R* represents the solution resistance and equals the ratio of *d*/*k* (*k* is the solution conductivity, 8.42 mS/cm for a 0.5% NaCl solution) [1,41]. The Δ*E* values of a region of 400 × 400 μm were recorded at the beginning of corrosion and after corrosion for 1 h, respectively. Therefore, the in-site distribution of the current density with corrosion time was obtained.

## 3. Results and Discussion

### 3.1. Corrosion Rate

**Figure 1:**
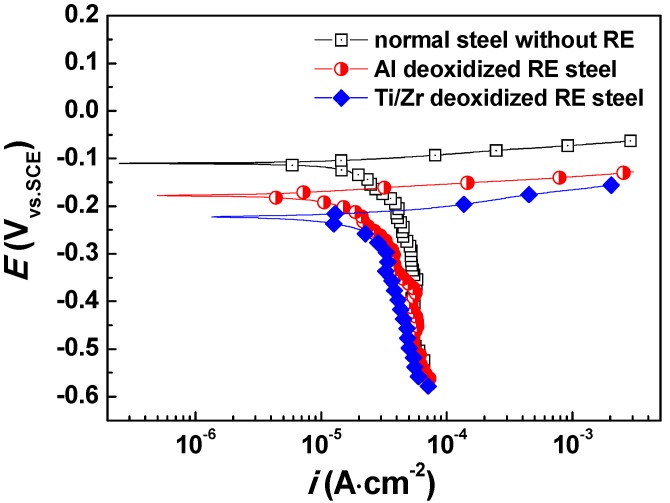
Potentiodynamic polarization curves of steels in a 3.5 wt.% NaCl solution.

**Figure 2:**
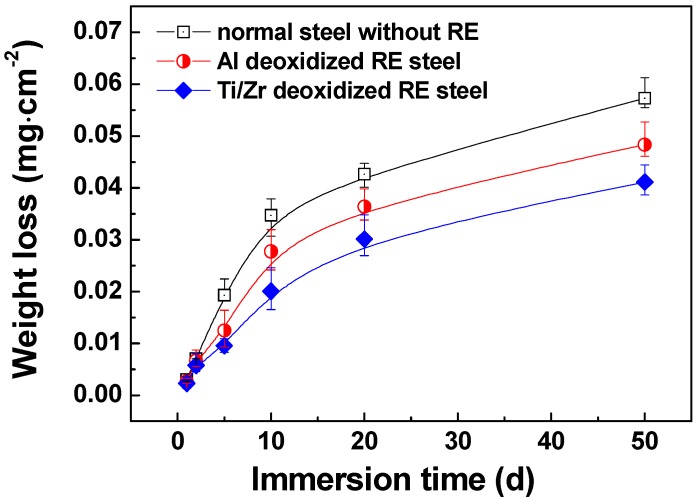
Corrosion weight loss of steels in a 3.5 wt.% NaCl solution.

In order to evaluate the corrosion rate of the microalloyed steels, potentiodynamic polarization tests were first carried out and the result is displayed in Figure 1 (in which, normal steel without a RE doping is also displayed for comparison). Moreover, the corresponding fitted corrosion current density (*i_corr_*) and corrosion potential (*E_corr_*) are shown in Table 2. The *i_corr_* of Ti/Zr deoxidized steel is lower than that of the Al deoxidized steel, and both of the RE microalloyed steels present lower corrosion rates than the normal steel without RE doping. In the long term corrosion experiment, as exhibited in Figure 2, the corrosion weight loss follows the same trend as those of potentiodynamic and potentiostatic polarizations. The corrosion weight loss decreases in the following order: Ti/Zr deoxidized steel < Al deoxidized steel < normal steel without RE doping. The weight loss increases with the immersion time, but the corrosion rate decreases as the immersion time prolongs.

### 3.2. Effect of RE Inclusions on Corrosion Nucleation Process

**Figure 3:**
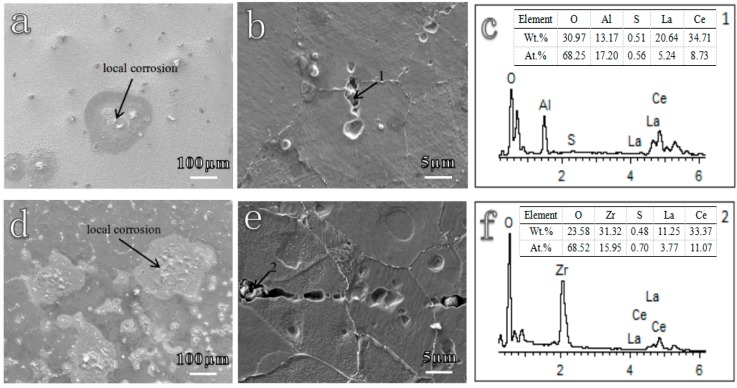
Corrosion morphologies of (**a**,**b**) Al deoxidized steel and (**d**,**e**) Ti/Zr deoxidized steel after immersion in 0.5 wt.% NaCl solution for a few minutes. (**a**,**d**) One hour, (**b**,**e**) 10 min, (**c**,**f**) EDS results of inclusions marked in **b** and **e**.

Figure 3 shows the corrosion morphologies of RE microalloyed steels after immersion in 0.5 wt.% NaCl solution for a few minutes. It could be observed from Figure 3a,d that corrosion originated from local corrosion, and then propagated to the whole surface for the two different deoxidized steels. Further observation showed that the local areas of corrosion origin were in the positions of inclusions, confirming that inclusions were the root of corrosion in microalloyed steels [8,9,10]. Therefore, it is necessary to identify the characteristic of inclusions to elucidate the original corrosion mechanism.

#### 3.2.1. Statistical Identification of Inclusions

SEM and EDS were employed to analyze the statistical characteristics of inclusions in a region of 30 mm^2^. The average distribution result is shown in Table 3. Several different types of sulfides, oxides, and oxygen sulfides have been reported to form in steels with the doping of RE elements [15,18,31,36,37,38,39,50,51,52]. Based on the experimental data, there are RE sulfides (RE-S_x_), RE oxides (RE-O_x_), Al mixed with RE oxides ((Al,RE)-O_x_), RE oxygen sulfides (RE-O_x_S_y_), and Al mixed with RE oxygen sulfides ((Al,RE)-O_x_S_y_) in Al deoxidized steel. For Ti/Zr deoxidized steel, there are RE-S_x_, RE-O_x_, Zr mixed with RE oxides ((Zr,RE)-O_x_), RE-O_x_S_y_, and Zr mixed with RE oxygen sulfides ((Zr,RE)-O_x_S_y_). It should be mentioned that the sulfides presented as RE-S_x_ exclusively, which could be ascribed to the more negative formation energy of RE-S_x_ than those of MnS and other metal sulfides [34,36,53]. Additionally, due to the strong affinity of RE with oxygen [37], a small quantity of Ti predominantly existed as TiC and TiN, but not as an oxide. Similarly, due to the low standard free energy for the formation of RE-O_x_ [25,34], the volume fractions of solitary Al_2_O_3_ and ZrO_2_ were less than 1%. Therefore, the aforementioned inclusions could be neglected in statistical analysis. In conclusion, the three main types of inclusions to be considered are metal sulfides (M-S_x_, referred to RE-S_x_), metal oxides (M-O_x_, referred to RE-O_x_, (Al,RE)-O_x_, and (Zr,RE)-O_x_), and metal oxygen sulfides (M-O_x_S_y_, referred to RE-O_x_S_y_, (Al,RE)-O_x_S_y_, and (Zr,RE)-O_x_S_y_), respectively.

**Figure 4:**
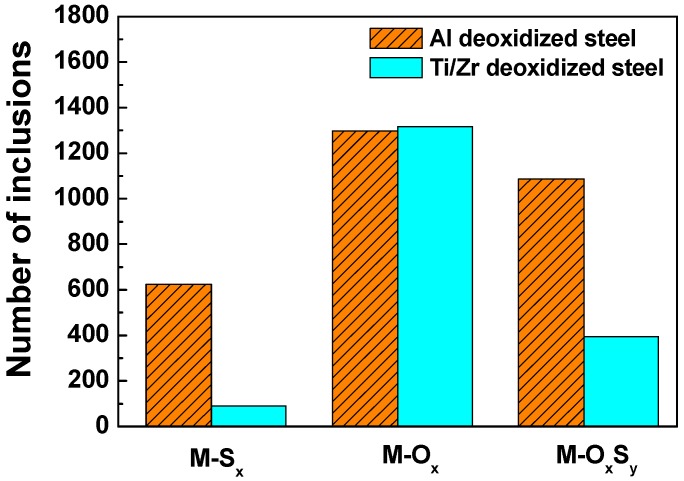
Number distributions of different kinds of inclusions in Al deoxidized steel and Ti/Zr deoxidized steel in a 30 mm^2^ scanning region.

**Figure 5:**
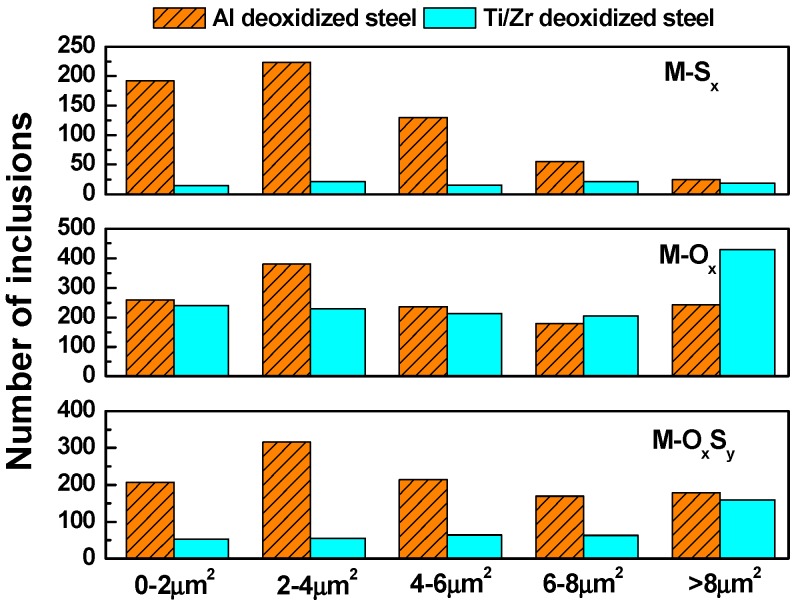
Size distributions of different kinds of inclusions in Al deoxidized steel and Ti/Zr deoxidized steel in a 30 mm^2^ scanning region.

It has been commonly recognized that not only the types of inclusions but also the size of inclusions has a great impact on the corrosion behavior [37]. Therefore, the number of distributions of different kinds of inclusions as well as the size of distributions in a 30 mm^2^ scanning region are depicted in Figure 4 and Figure 5, respectively. The overall numbers of inclusions are 3009 and 1801 for Al deoxidized steel and Ti/Zr deoxidized steel, respectively. The metal oxide (M-O_x_) inclusions are the majority in both steels. However, the amount of metal sulfides (M-S_x_) and metal oxygen sulfides (M-O_x_S_y_) in Al deoxidized steel is obviously higher than that in Ti/Zr deoxidized steel (Figure 4). Additionally, according to the size distributions in Figure 5, regardless of the type of inclusions, the amount of inclusions in Al deoxidized steels is higher than that in Ti/Zr deoxidized steels at almost each size range, except for the metal oxides (M-O_x_) with a particle size larger than 6 µm. In general, the Al deoxidized steels contain more inclusions with larger particle sizes.

#### 3.2.2. Corrosion Behavior of Inclusions

**Figure 6:**
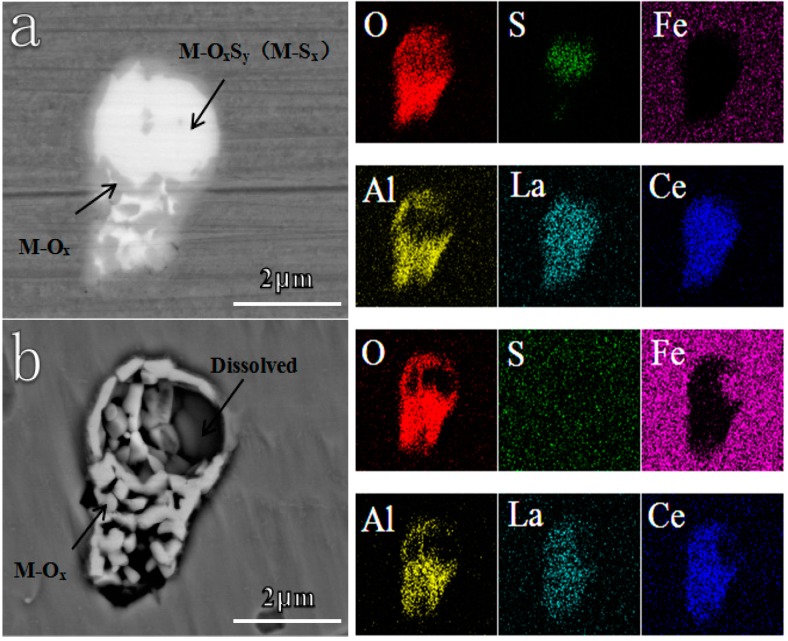
In-situ dissolution behavior of the inclusion in Al deoxidized steel after immersion in 0.5 wt.% NaCl for 10 min. (**a**) Before corrosion, (**b**) after corrosion.

Subsequently, the corrosion behaviors of inclusions in different deoxidized steels were characterized. For most cases, two or more components coexisted in one inclusion, which has been reported in previous studies [37,53,54,55]. As seen in Figure 6, the light part in Al deoxidized steel is denoted as metal oxygen sulfides and/or metal sulfides, M-O_x_S_y_ (M-S_x_), while, the dark part is metal oxides, M-O_x_, which could be verified by the EDS results. After immersion in a 0.5 wt.% NaCl solution for 10 min, parts of M-O_x_S_y_ (M-S_x_) became voids. Correspondingly, the concentration of O, S, and RE elements reduced, while the Fe content increased in that original region, indicating the complete dissolution of M-O_x_S_y_ (M-S_x_). In contrast, there was no change in the state of M-O_x_.

**Figure 7:**
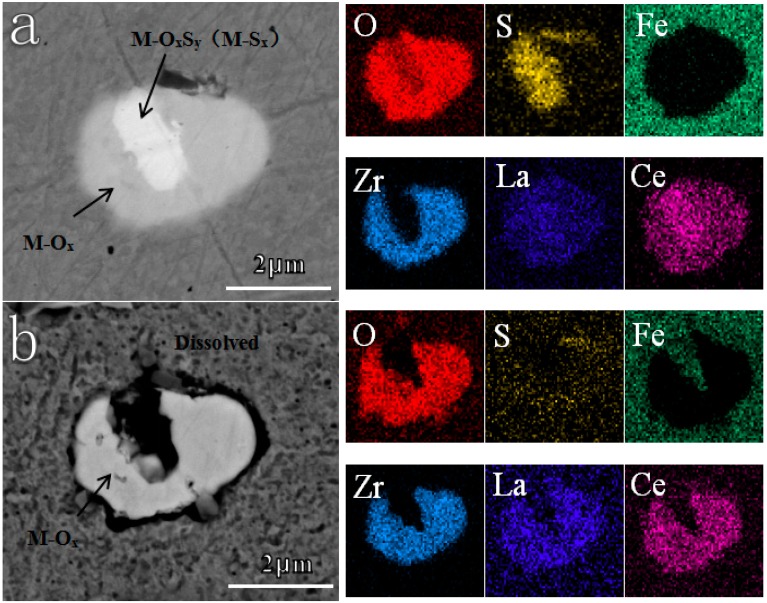
In-situ dissolution behavior of the inclusion in Ti/Zr deoxidized steel after immersion in 0.5 wt.% NaCl for 10 min. (**a**) Before corrosion, (**b**) after corrosion.

With respect to Ti/Zr deoxidized steel, two or more components also coexisted in one inclusion for most cases (Figure 7). One is M-O_x_S_y_ (M-S_x_), and the other is M-O_x_. After immersion for 10 min, EDS mapping revealed that O and S, as well as some RE elements, disappeared in part of M-O_x_S_y_ (M-S_x_). Correspondingly, the Fe matrix became bare. The M-O_x_S_y_ (M-S_x_) dissolved completely. However, the M-O_x_ did not dissolve at all. Additionally, there was slight corrosion at the boundary between the Fe matrix and oxides.

**Figure 8:**
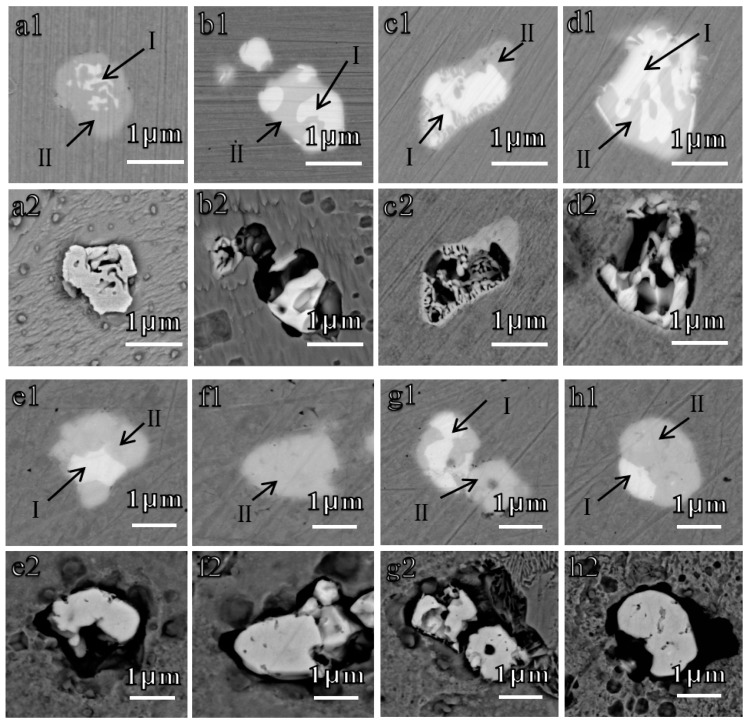
In-situ dissolution behaviors of inclusions in (**a**–**d**) Al deoxidized steel and (**e**–**h**) Ti/Zr deoxidized steel after immersion in 0.5 wt.% NaCl for 10 min. (**a1**–**h1**) Before corrosion, (**a2**–**h2**) after corrosion. The inclusions marked Ι are M-O_x_S_y_ (M-S_x_) and ΙΙ are M-O_x_, which are the same as those in Figure 6 and Figure 7.

There were many other similar examples manifesting this dissolution phenomenon, as shown in Figure 8. The inclusions exhibited the same dissolution behavior as aforementioned. M-O_x_S_y_ and/or M-S_x_ dissolved entirely after immersion in NaCl solution for 10 min, whereas, M-O_x_ remained. The corrosion also occurred at the boundary between the Fe matrix and oxides for both steels.

**Figure 9:**
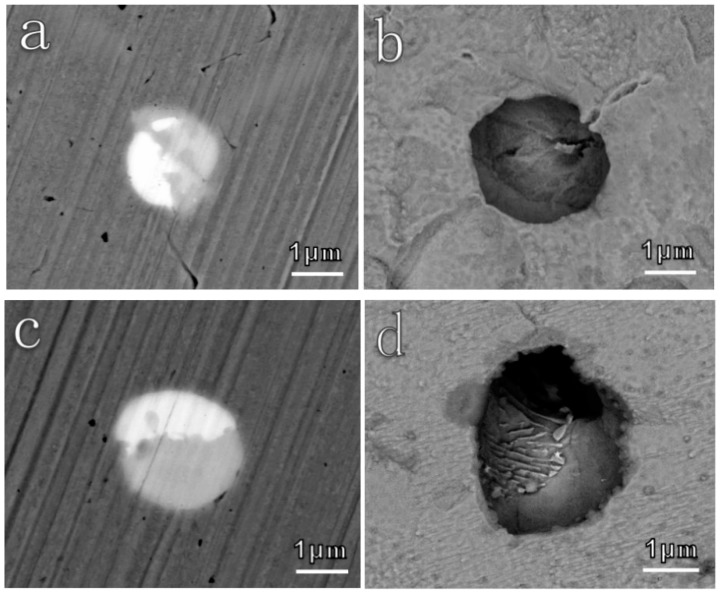
In-situ dissolution morphologies of inclusions in (**a**,**b**) Al deoxidized steel and (**c**,**d**) Ti/Zr deoxidized steel after immersion in 0.5 wt.% NaCl solution for 30 min. The left is before corrosion, and the right is after corrosion.

In conclusion, the dissolution behaviors of the inclusions can be summarized as follows: First, M-O_x_S_y_ (M-S_x_) dissolved preferentially, while M-O_x_ did not undergo any dissolution [21,35,36]. Afterwards, slight corrosion occurred at the boundary between the Fe matrix and M-O_x_ [36,37,56], which finally led to the complete fall off of the inclusions [37]. Figure 9 shows the dissolution morphologies of the inclusions after immersion in a NaCl solution for 30 min, exhibiting obvious voids in the locations of the original inclusions. The voids due to the overall fall off of the inclusion, acting as the active sites, promoted pitting nucleation [37]. Considering the high amount and large size of M-S_x_ and M-O_x_S_y_ in Al deoxidized steel, there were more active sites on its surface. Therefore, the Al deoxidized steel presented a higher corrosion rate than Ti/Zr deoxidized steels.

#### 3.2.3. Theoretical Explanation for the Corrosion Behavior of Inclusions

Based on the above experimental results, first principle modeling was used to calculate work functions to theoretically explain the dissolution behavior of inclusions. Work function equals the difference between the vacuum static potential and Fermi energy, which is the lowest energy needed for an electron to escape from a material surface, and reflects the corrosion tendency of the material itself. In general, low-indexed crystallographic planes with relatively lower surface energy have a higher probability of becoming the exposed surface, which easily participates in the electrochemical corrosion process [41]. Therefore, the vacuum static potentials, Fermi energies, and work functions of the low-indexed crystallographic planes, such as the (100), (110), and (111) planes of the various inclusions, were calculated to evaluate the corrosion potential of these inclusions [41,56].

**Figure 10:**
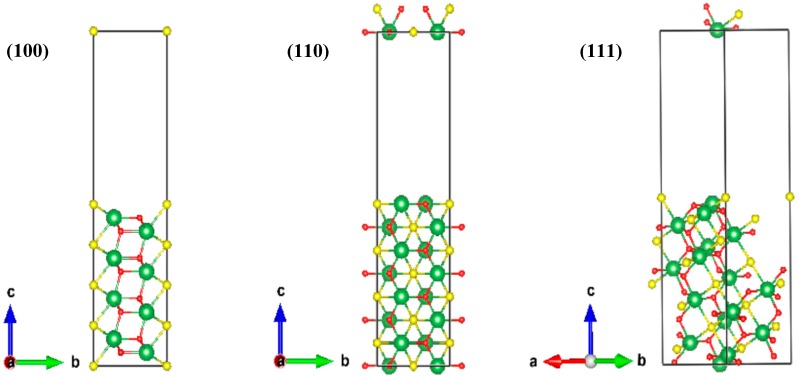
Slab models of the three low-indexed crystallographic planes of La_2_O_2_S. Green balls refer to La elements, yellow balls refer to S elements, and red balls refer to O elements. The left is (100) crystallographic planes, the middle is (110) crystallographic planes and the right is (111) crystallographic planes of La_2_O_2_S. The arrows of a, b, and c refer to the three directions in rectangular coordinates.

According to the statistical results of inclusions (Table 3), the inclusions can be divided into three groups, that is, metal sulfides M-S_x_, metal oxides M-O_x_, and metal oxygen sulfides M-O_x_S_y_, respectively. Considering the complexity for Ce to achieve the convergence during the characteristic calculation of Ce inclusions, La was chosen as the representation of the RE element for its stability [36]. Therefore, the existing form of M-S_x_ is represented by La_2_S_3_, and M-O_x_S_y_ refers to La_2_O_2_S according to previous studies [33,37,41]. With respect to M-O_x_, there are La_2_O_3_, LaAlO_3_, and La_2_Zr_2_O_7_ as the commonly existing forms for (Al,RE)-O_x_ and (Zr,RE)-O_x_ [33,36]. The slab models of three low-indexed crystallographic planes of the aforementioned inclusions were set up, for example, La_2_O_2_S was set to show the establishment of the model and presented in Figure 10. Finally, the results of vacuum static potentials, Fermi energies, and work functions of the aforementioned inclusions are displayed in Table 4, Table 5 and Table 6, respectively. The descending order of work functions were followed by: La_2_Zr_2_O_7_ > LaAlO_3_ > (La_2_O_3_ ≈ Fe ≈ La_2_O_2_S) > La_2_S_3_. This result explains the experimental results theoretically, suggesting that sulfides (oxygen sulfides) tend to dissolve preferentially in contrast to the Fe matrix, while oxides are not easy to dissolve, which is consistent with previous experimental studies [21,25,35,36,37].

### 3.3. Effect of RE Inclusions on the Corrosion Propagation

**Figure 11:**
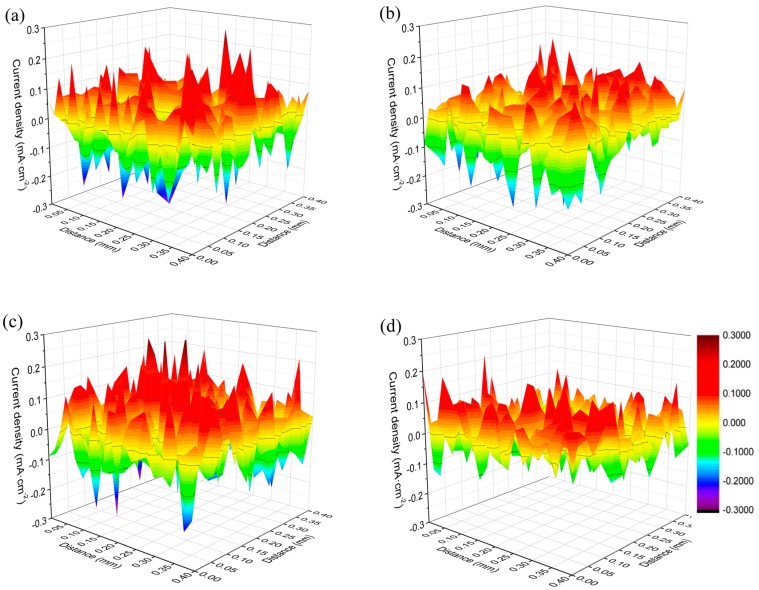
SVET images of the local current distributions on the metal surface of (**a**,**b**) Al deoxidized steels and (**c**,**d**) Ti/Zr deoxidized steels after immersion in 0.5 wt.% NaCl solution for different times. (**a**,**c**) Immersed for 10 min, (**b**,**d**) immersed for 1 h.

SVET provides a more microscopic insight to characterize the in-situ microelectrochemical activities on the metal surface, which will establish an in-depth mechanistic understanding of the pitting propagation process [1]. In present work, SVET was employed to analyze the distribution of the current density for the two different deoxidized RE steels at the beginning of exposure (10 min) and after 1 h exposure in a 0.5 wt.% NaCl solution. The SVET mappings are shown in Figure 11. It can be seen that these maps show clear differences between the anodic and cathodic sites at the surface [47]. The more severe the current fluctuation, the greater the intensity of local corrosion. For the initial exposed specimens in Figure 11a,c, there were lots of ups and downs in the current on the metal surface, illustrating that the pitting of inclusions on the surface brought out great nonuniformity of the electrochemical activity. After corrosion for 1 h, the fluctuation tendency of the current evened out (Figure 11b,d), which suggested that the difference in the electrochemical activity among local sites was reduced. Therefore, it can be inferred that active sites of pitting nucleation accelerated the transverse propagation of corrosion, as illustrated in the SVET results that the electrochemical activity tended to be uniform. As a result, local corrosion spread to the whole surface as uniform corrosion in the later corrosion process.

**Figure 12:**
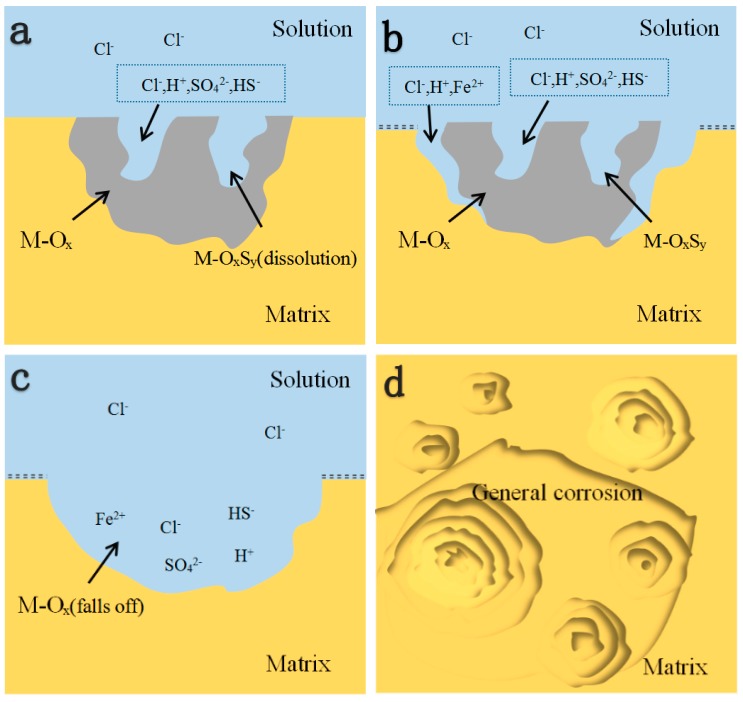
Schematic diagram of the corrosion process of RE microalloyed steels from pitting nucleation (**a**), to pitting growth (**b**,**c**), further to the propagation of general corrosion (**d**).

A simple schematic diagram for the role of inclusions in the corrosion process is depicted in Figure 12. The corrosion process consists of a successive series of pitting nucleation (dissolution of sulfides, Figure 12a), pitting growth (fall off of inclusions as a whole, Figure 12b,c), and propagation of general corrosion (transverse spread of the active sites, Figure 12d). The corrosion process can be understood as follows. In the initial stage, the inclusions containing sulfur dissolved preferentially due to the lower values of work functions of M-S_x_ and M-O_x_S_y_. As the sulfides dissolved, the acid solution accumulated in the pit nucleus, promoting further dissolution of the surrounding metals or oxides [37]. Subsequently, the inclusion fell off as a whole due to the dissolution of the boundary between the Fe matrix and oxides [37]. Finally, with the propagation and transverse spread of pitting nuclei, the local corrosion tended to become uniform corrosion. From the theoretical and experimental perspective, the modeling of the first principle and the measurement of SVET provided more microscopic and more in-depth insight into the role of inclusions in the initial marine corrosion process of microalloyed steels.

## 4. Conclusions

The role of rare earth inclusions in the initial marine corrosion process of microalloyed steels could be summarized as follows:

(1) The corrosion of RE microalloyed steels originated from inclusions, which contained metal sulfides, metal oxides, and metal oxygen sulfides. The metal sulfides and/or metal oxygen sulfides tended to dissolve preferentially, whereas the metal oxides remained the same. With the dissolution of the Fe matrix–oxides interface, the inclusions fell off as a whole, which promoted pitting nucleation.

(2) The work functions of various kinds of inclusions were calculated by first principle modeling. The descending order of work functions were followed by: La_2_Zr_2_O_7_ > LaAlO_3_ > (La_2_O_3_ ≈ Fe ≈ La_2_O_2_S) > La_2_S_3_, which provided a qualitative explanation to the dissolution behaviors of inclusions. That is, inclusions containing sulfur tended to dissolve preferentially, whereas the oxides were not easy to dissolve.

(3) SVET provided a promising method to characterize the micro-electrochemical activities on the surface. The surface current distributions illustrated that the transverse propagation of corrosion promoted the formation of uniform corrosion throughout the surface.

(4) With respect to the effect of different deoxidization methods, the Ti/Zr deoxidized RE microalloyed steel presented better corrosion resistance than Al deoxidized steel, which could be ascribed to the lower size and quantity of RE sulfides in Ti/Zr deoxidized steel.

## Figures and Tables

**Table 1:** Chemical compositions of the RE microalloyed steels (wt.%).

Sample	C	Si	Mn	P	S	O	Cu	Cr	Ni	Al	Ti	Zr	La	Ce	Fe
Al deoxidized	0.053	0.23	0.13	0.005	0.003	0.050	0.39	1.21	0.31	0.03	-	-	0.002	0.003	balance
Ti/Zr deoxidized	0.050	0.22	0.13	0.005	0.003	0.065	0.43	1.22	0.33	-	0.008	0.008	0.002	0.003	

**Table 2:** Corrosion current density and corrosion potential obtained from potentiodynamic polarization curves in Figure 1.

Sample	*i_corr_* (×10^−5^ A·cm^−2^)		*E_corr_* (V)	
	Mean	Deviation	Mean	Deviation
Normal steel without RE	5.31	±0.46	−0.122	±0.013
Al deoxidized RE steel	4.60	±0.33	−0.137	±0.010
Ti/Zr deoxidized RE steel	2.93	±0.60	−0.237	±0.023

**Table 3:** Statistical distributions of inclusions in RE microalloyed steels.

Sample	Types	Density (mm^−2^)		Average Size (µm^2^)	
		Individual	Overall	Individual	Overall
Al deoxidized	RE-S_x_	20.81	100.29	3.36	4.02
	RE-O_x_	14.95		3.81	
	(Al,RE)-O_x_	28.31		4.15	
	RE-O_x_S_y_	18.34		3.98	
	(Al,RE)-O_x_S_y_	17.88		4.78	
Ti/Zr deoxidized	RE-S_x_	2.97	60.04	5.85	6.11
	RE-O_x_	3.52		5.13	
	(Zr,RE)-O_x_	40.38		5.83	
	RE-O_x_S_y_	3.75		6.17	
	(Zr,RE)-O_x_S_y_	9.42		7.57	

**Table 4:** Vacuum static potentials of inclusions and the Fe matrix at three different low-indexed crystallographic planes (eV).

Crystallographic Plane	La_2_S_3_	La_2_O_3_	LaAlO_3_	La_2_Zr_2_O_7_	La_2_O_2_S	Fe
(100)	5.20	7.32	7.06	6.70	6.14	5.64
(110)	5.20	7.87	7.37	6.07	5.55	6.79
(111)	5.48	5.77	5.96	6.06	5.59	4.02

**Table 5:** Fermi energies of inclusions and the Fe matrix at three different low-indexed crystallographic planes (eV).

Crystallographic Plane	La_2_S_3_	La_2_O_3_	LaAlO_3_	La_2_Zr_2_O_7_	La_2_O_2_S	Fe
(100)	2.07	2.46	2.55	0.12	−0.21	1.10
(110)	1.50	2.91	1.82	0.38	1.80	1.85
(111)	1.27	2.17	2.23	−0.63	1.87	−0.30

**Table 6:** Work functions of inclusions and the Fe matrix at three different low-indexed crystallographic planes (eV).

Crystallographic Plane	La_2_S_3_	La_2_O_3_	LaAlO_3_	La_2_Zr_2_O_7_	La_2_O_2_S	Fe
(100)	3.13	4.86	4.51	6.58	6.35	4.54
(110)	3.70	4.96	5.32	5.69	3.75	5.12
(111)	4.21	3.60	3.73	6.70	3.72	4.32
Average	3.68	4.47	4.52	6.32	4.61	4.66
